# Primary Treatment Modification and Treatment Tolerability Among Older Chemotherapy Recipients With Advanced Cancer

**DOI:** 10.1001/jamanetworkopen.2023.56106

**Published:** 2024-02-15

**Authors:** Mostafa R. Mohamed, David Q. Rich, Christopher Seplaki, Jennifer L. Lund, Marie Flannery, Eva Culakova, Allison Magnuson, Megan Wells, Rachael Tylock, Supriya G. Mohile

**Affiliations:** 1 Division of Hematology/Oncology, James P. Wilmot Cancer Institute, Department of Medicine, University of Rochester, Rochester, New York; 2 Department of Public Health, University of Rochester School of Medicine and Dentistry, Rochester, New York; 3 Department of Epidemiology, University of North Carolina, Chapel Hill; 4 University of Rochester School of Nursing, Rochester, New York; 5 Department of Surgery, University of Rochester School of Medicine and Dentistry, Rochester, New York

## Abstract

**Importance:**

Older adults with advanced cancer are less likely to tolerate treatment with cytotoxic chemotherapy compared with younger patients due to their aging-related conditions. Hence, oncologists sometimes opt to employ primary treatment modifications (deviation from standard of care) during the first cycle of chemotherapy.

**Objective:**

To examine the association between primary treatment modification and treatment tolerability in older adults with advanced cancer who were starting new palliative chemotherapy regimens.

**Design, Setting, and Participants:**

This cohort study was a secondary analysis of the GAP70+ (Geriatric Assessment Intervention for Reducing Toxicity in Older Patients with Advanced Cancer) trial, which was conducted between July 2014 and March 2019. The GAP70+ trial included patients aged 70 years or older who had advanced (ie, incurable) cancer, had 1 or more geriatric assessment domain impairments, and planned to start a new palliative chemotherapy regimen. Data analysis was conducted in November 2022.

**Exposures:**

Receipt of standard-of-care chemotherapy regimens vs primary treatment modification defined as any change from National Comprehensive Cancer Network guidelines or published clinical trials (eg, primary dose reduction, schedule change).

**Main Outcomes and Measures:**

Tolerability outcomes were assessed within 3 months of treatment. These outcomes included the following: (1) any grade 3 to 5 toxic effect, according to the National Cancer Institute Common Terminology Criteria for Adverse Events; (2) patient-reported functional decline, defined as the development of worse dependency in activities of daily living using scale scores; and (3) a composite adverse outcome (an end point that combined toxic effects, functional decline, and 6-month overall survival). Multivariable cluster-weighted generalized estimating equation models examined the association between primary treatment modification and outcomes adjusting for covariates.

**Results:**

This study included 609 patients with a mean (SD) age of 77.2 (5.2) years; more than half (333 [54.7%]) were men. Race and ethnicity was available for 607 patients: 39 (6.4%) were Black, 539 (88.5%) were non-Hispanic White, and 29 (4.8%) were of other race or ethnicity. Nearly half (281 [46.1%]) received a primary modified treatment regimen. The most common cancer types were gastrointestinal cancer (228 [37.4%]) and lung cancer (174 [28.6%]). In multivariable analysis, primary treatment modification was associated with a reduced risk of grade 3 to 5 toxic effects (relative risk [RR], 0.85 [95% CI, 0.77-0.94]) and functional decline (RR, 0.80 [95% CI, 0.67-0.95]). Patients who received primary treatment modification had 32.0% lower odds of having a worse composite adverse outcome (odds ratio, 0.68 [95% CI, 0.48-0.97]).

**Conclusions and Relevance:**

In this cohort study, primary treatment modification was associated with improved tolerability of chemotherapeutic regimens among older adults with advanced cancer and aging-related conditions. These findings may help optimize cancer treatment dosing in older adults with advanced cancer and aging-related conditions.

## Key Points

**Question:**

What is the association between primary treatment modification (eg, primary dose reduction and schedule modification) and tolerability outcomes in older adults with advanced cancer?

**Findings:**

In this cohort study of 609 older adults with advanced cancer who were starting a new chemotherapy regimen in the community oncology setting, patients who had primary treatment modification had a 15% reduced risk of serious clinician-rated toxic effects and a 20% reduced risk of patient-reported functional decline compared with those who received standard doses.

**Meaning:**

These findings suggest that receiving primary treatment modification may lead to better tolerability of chemotherapeutic regimens among older adults with advanced cancer and aging-related conditions.

## Teaser

This cohort study evaluates the association between primary treatment modification and treatment tolerability in older adults with advanced cancer who were starting new palliative chemotherapy regimens in the community oncology setting.

## Introduction

Older adults are disproportionately underrepresented in cancer trials that are used to establish treatment guidelines.^1,2,3^ Hence, the reported risks and benefits of cancer treatment are based on clinical trials conducted in younger and healthier patients.^4,5^ This underrepresentation results in uncertainties about the standard-of-care treatments for older patients, including treatment safety and efficacy for older adults who have aging-related conditions.

Older adults with cancer are generally considered to be more vulnerable to the adverse effects of cytotoxic chemotherapy (eg, poor tolerability) compared with younger patients,^6^ due to disabilities, deterioration in organ function, or other geriatric impairments (eg, impaired cognition) that may add to treatment toxicity.^6,7,8^ Despite these vulnerabilities, older adults are often given aggressive chemotherapeutic agents with a high risk of toxicity (eg, febrile neutropenia, fatigue, and neuropathy), which can ultimately lead to treatment delays and poor cancer control.^6,9^

Although aggressive cytotoxic therapies have the potential to extend life among older adults with advanced cancer and aging-related conditions, they also can cause serious adverse events that may worsen quality of life.^6^ Due to unclear data on treatment decision-making in older adults with advanced cancer, oncologists may modify treatment during the first cycle of chemotherapy (ie, primary treatment modification) by dose reduction, schedule alteration, or use of less toxic regimens to reduce the potential side effects of systemic chemotherapy.^10^ However, the side effects of these modifications on both tolerability and efficacy outcomes in older adults with advanced cancer are understudied.

In the advanced cancer setting, in which the primary objective of treatment is palliation, oncologists strive to control the disease while preserving physical function, and patients are frequently compelled to make trade-offs between treatment tolerability and survival.^11^ Hence, it is important to construct composite measures that concurrently assess both tolerability and efficacy end points. This approach could provide a more comprehensive evaluation of the beneficial and detrimental effects of cancer treatments.

In this study, we extend our previous findings suggesting that increased chronological age, poor functional status, and lower annual income are associated with primary treatment modification among older adults with advanced cancer receiving systemic treatment.^12^ We aimed (1) to estimate the associations of primary treatment modification with tolerability outcomes (serious toxic effects as rated by clinicians, and patient-reported functional decline) and (2) to evaluate the association between primary treatment modification and a composite adverse outcome (clinician-rated serious toxic effects, patient-reported functional decline, and 6-month overall survival). We hypothesized that older adults with advanced cancer who had primary treatment modification would have a lower risk of poor tolerability compared with older adults with advanced cancer who received standard-of-care regimens.

## Methods

### Study Design and Participants

This cohort study was a secondary analysis of the completed nationwide, multicenter, cluster-randomized GAP70+ (Geriatric Assessment Intervention for Reducing Toxicity in Older Patients with Advanced Cancer) study.^13^ The GAP70+ study was conducted by the University of Rochester Cancer Control (URCC) National Cancer Institute (NCI) Community Oncology Research Program (NCORP) Research Base and approved by the institutional review boards at participating sites (40 community oncology practice clusters across the US). All patient participants provided written informed consent. A waiver of documentation of consent was approved for clinicians and staff. This analysis followed the Strengthening the Reporting of Observational Studies in Epidemiology (STROBE) reporting guideline for cohort studies.

The GAP70+ study assessed whether providing information regarding geriatric assessment (GA) management to community oncologists reduced clinician-rated serious chemotherapy toxic effects in older adults with advanced cancer who were starting a new cancer treatment regimen between July 2014 and March 2019.^13^ Eligible patients for the current analysis were (1) aged 70 years or older; (2) diagnosed with an incurable stage III or IV solid tumor or lymphoma; (3) had impairment in at least 1 GA domain; and (4) were planning to start a new cytotoxic chemotherapy regimen within 4 weeks of enrollment.

### Primary Exposure

Primary treatment modification at the beginning of the chemotherapy course was defined as any change in dose or agents of the planned regimen from the standard (National Comprehensive Cancer Network [NCCN]) treatment guidelines^14^ or published phase 2 or 3 clinical trials. Regimens that met the standard guidelines for dose and scheduling were defined as the standard dose.

All of the planned regimens (individual drugs, doses, and schedule) were captured at the beginning of the GAP70+ study from the primary oncology team and reviewed by a blinded research team at the URCC NCORP Research Base, including at least 2 oncology clinicians (M.R.M., M.F., A.M., or S.G.M.) and an information analyst. For each participant, modifications were classified as follows: (1) dose reduction (ie, chemotherapy dose lower than the established regimen), (2) modified schedule to administer chemotherapy less frequently than the established regimen, or (3) modified regimen (eg, ≥1 agents were intentionally left out of a polychemotherapy regimen). The composite binary variable of primary treatment modification (nonstandard vs standard) was constructed as the presence of any of these types of treatment modifications and served as the primary independent variable in analyses.

### Study Outcomes

The primary outcome was grade 3 to 5 toxic effects (as measured with the NCI Common Terminology Criteria for Adverse Events, version 4) within 3 months of starting a new chemotherapy regimen.^15^ The process of toxicity ascertainment is described in detail in the parent study.^13^

The secondary outcomes were (1) patient-reported functional decline and (2) the composite adverse outcome. Patient-reported functional decline was defined as the development of new or worse dependency in activities of daily living (ADL) according to ADL index scores.^16^ Activities of daily living were measured before initiation of treatment and again after 3 months of treatment. Patients were asked whether they needed assistance with bathing, dressing, walking, getting in or out of bed or chairs, toileting, or eating. A decrease of 1 point on the scale between measurements made at the time of GAP70+ study enrollment and at 3 months was considered clinically relevant (ie, presence of ADL functional decline).^17^ In addition to measuring functional decline using ADLs, we conducted an exploratory analysis to assess the association between primary treatment modification and declines in more complex activities, specifically instrumental activities of daily living (IADLs). Instrumental activities of daily living encompass activities related to independent living and are considered more complex than basic self-care ADLs.^18^ A decrease of 1 point on the scale between measurements made at the time of GAP70+ study enrollment and at 3 months was considered clinically relevant.

The composite adverse outcome measure incorporated both clinician-rated toxic effects and patient-reported functional decline with survival. A favorable composite adverse outcome was defined as follows: (1) no grade 3 or greater toxic effects, (2) no patient-reported functional decline, and (3) no mortality within 6 months of enrollment. An intermediate composite adverse outcome was defined as (a) poor tolerability through the presence of either any grade 3 or greater toxic effect or patient-reported functional decline or (b) mortality within 6 months of treatment. An unfavorable composite adverse outcome was defined as (a) the presence of poor tolerability (presence of any grade ≥3 toxic effect or of patient-reported functional decline) and (b) mortality within 6 months of the start of treatment. We considered 6-month mortality because it identifies older persons at risk of early mortality after beginning chemotherapy.^19,20^ Because the recent NCCN treatment guidelines support early palliative care, advance care planning, and therapy arrangement according to life expectancy, it is crucial for oncology clinicians to identify patients at high risk of short-term mortality (ie, mortality within 6 months), which indicates that chemotherapy may cause toxicity and may not result in long-term survival benefits.^20,21^

### Covariates

The GAP70+ study included an extensive set of covariates that were all recorded before initiation of the chemotherapy regimen, including variables associated with poor tolerability among older adults with cancer receiving chemotherapy.^22,23^ These variables included the following: (1) sociodemographic variables, including age in years, sex, race and ethnicity, education, and income; (2) baseline clinical variables, including cancer type, cancer stage (III, IV, or other), line of treatment, multiple chemotherapy agents (yes or no), and physician-reported Karnofsky Performance Scale score (ordinal from 20 to 100)^24^; and (3) GA variables (described in detail in the parent study^13^ and in eTable 1 in Supplement 1). Analysis of race and ethnicity data was deemed relevant to acknowledge potential disparities in representation between groups. These data were reported as Black, non-Hispanic White (hereinafter, White), or other race or ethnicity (American Indian or Alaska Native, Asian, or Native Hawaiian or Other Pacific Islander).

### Statistical Analysis

We generated descriptive statistics to summarize baseline variables, primary treatment modification, and outcome measures (overall and across different cancer types). To determine whether primary treatment modification was associated with tolerability outcomes (grade 3-5 toxic effects and functional decline), we conducted both unadjusted and adjusted analyses. We used a cluster-weighted generalized estimating equation model with a binary distribution, log link, and robust SEs. This model accounts for correlations among patients from the same practice. Generalized estimating equation models were built using a manual backward selection method. In multivariable adjusted analyses, the parent study group was a priori included in all models because the primary study demonstrated that GA intervention reduced toxic effects from cancer treatment.^13^ Additional covariates were determined based on bivariate analyses, with a threshold of *P* < .10 (2-tailed), identifying sociodemographic and clinical factors that had potential associations with outcomes. This same model-building process was used for each of the other outcome measures.

To examine the association between primary treatment modification and levels of the composite adverse outcome (ie, favorable, intermediate, or unfavorable), we constructed a proportional odds model adjusting for the same covariates using the selection approach just described. *P* < .05 (2-tailed) defined statistical significance. All analyses were conducted using SAS, version 9.4 (SAS Institute Inc). Data analysis was conducted in November 2022.

## Results

### Study Population Characteristics

The study schema for the 609 participants included in this analysis is described in eFigure 1 in Supplement 1. Their mean (SD) age was 77.2 (5.2) years. Overall, 333 (54.7%) were men and 275 (45.2%) were women (sex was missing for 1 participant). Race and ethnicity data were available for 607 patients: 39 (6.4%) were Black, 539 (88.5%) were White, and 29 (4.8%) were of other race or ethnicity. The majority of patients (526 [86.4%]) had stage IV cancer. The most common cancer types were gastrointestinal cancer (228 [37.4%]) and lung cancer (174 [28.6%]). At the time of enrollment, 458 patients (75.2%) were scheduled to receive first-line palliative chemotherapy. Other patient characteristics are outlined in Table 1, and the most common treatment regimens are detailed in Table 2. Of the 609 patients, 281 (46.1%) received a modified treatment regimen. The most common forms of primary treatment modification were primary dose reduction (202 [71.9%]), followed by schedule change (33 [11.7%]; eFigure 2 in Supplement 1).

**Table 1.** Characteristics of Patients Receiving Primary Treatment Modification vs Standard-of-Care Regimens^a^

Characteristic	All patients (N = 609)		Cancer type					
			Gastrointestinal (n = 228)		Lung (n = 174)		Other (n = 207)^b^	
	Primary treatment modification (n = 281)	Standard of care (n = 328)	Primary treatment modification (n = 107)	Standard of care (n = 121)	Primary treatment modification (n = 77)	Standard of care (n = 97)	Primary treatment modification (n = 97)	Standard of care (n = 110)
Age, y								
70-79	177 (63.0)	249 (75.9)	70 (65.4)	94 (77.7)	53 (68.8)	80 (82.5)	54 (55.7)	75 (68.2)
≥80	103 (36.7)	79 (24.1)	36 (33.6)	27 (22.3)	24 (31.2)	17 (17.5)	43 (44.3)	35 (31.8)
Sex (n = 608)								
Male	147 (52.3)	186 (56.7)	56 (52.3)	66 (54.6)	43 (55.8)	63 (65.0)	48 (49.5)	57 (51.8)
Female	133 (47.3)	142 (43.3)	50 (46.7)	55 (45.5)	34 (44.2)	34 (35.1)	49 (50.5)	53 (48.2)
Race (n = 607)								
Black	21 (7.5)	18 (5.5)	11 (10.3)	7 (5.8)	1 (1.3)	6 (6.2)	9 (9.3)	5 (4.6)
Non-Hispanic White	244 (86.8)	295 (89.9)	87 (81.3)	108 (89.3)	71 (92.2)	88 (90.7)	86 (88.7)	99 (90.0)
Other^c^	14 (5.0)	15 (4.6)	7 (6.5)	6 (5.0)	5 (6.5)	3 (3.1)	2 (2.1)	6 (5.5)
Education								
Less than high school	49 (17.4)	48 (14.6)	20 (18.7)	16 (13.2)	15 (19.5)	20 (20.6)	14 (14.4)	12 (10.9)
High school	102 (36.3)	106 (32.3)	42 (39.3)	34 (28.1)	25 (32.5)	33 (34.0)	35 (36.1)	39 (35.4)
College or above	129 (45.9)	174 (53.1)	44 (41.1)	71 (58.7)	37 (48.1)	44 (45.4)	48 (49.5)	59 (53.6)
Income, $								
≤50 000	156 (55.5)	165 (50.3)	61 (57.0)	58 (47.9)	46 (59.7)	52 (53.6)	49 (50.5)	55 (50.0)
>50 000	59 (21.0)	93 (28.4)	22 (20.6)	31 (25.6)	13 (16.9)	24 (24.7)	24 (24.7)	38 (34.6)
Declined to answer	65 (23.1)	70 (21.3)	23 (21.5)	32 (26.5)	18 (23.4)	21 (21.6)	24 (24.7)	17 (15.5)
Cancer type								
Gastrointestinal	107 (38.1)	121 (36.9)	NA	NA	NA	NA	NA	NA
Genitourinary	29 (10.3)	26 (7.9)	NA	NA	NA	NA	NA	NA
Gynecologic	21 (7.5)	18 (5.5)	NA	NA	NA	NA	NA	NA
Breast	15 (5.3)	22 (6.7)	NA	NA	NA	NA	NA	NA
Lung	77 (27.4)	97 (29.6)	NA	NA	NA	NA	NA	NA
Lymphoma	11 (3.9)	33 (10.1)	NA	NA	NA	NA	NA	NA
Other	21 (7.5)	11 (3.4)	NA	NA	NA	NA	NA	NA
Cancer stage								
III	36 (12.8)	36 (11.0)	7 (6.5)	13 (10.7)	17 (22.1)	12 (12.4)	12 (12.4)	11 (10.0)
IV	242 (86.1)	284 (86.6)	99 (92.5)	106 (87.6)	59 (76.4)	84 (86.6)	84 (86.6)	94 (85.5)
Other	3 (1.1)	8 (2.4)	1 (0.9)	2 (1.7)	1 (1.3)	1 (1.0)	1 (1.0)	5 (4.6)
KPS score								
20-60	43 (15.3)	34 (10.4)	17 (15.9)	13 (10.7)	13 (16.9)	14 (14.4)	13 (13.4)	7 (6.4)
70-100	238 (84.7)	294 (89.6)	90 (84.1)	108 (89.3)	64 (83.1)	83 (85.6)	84 (86.6)	103 (93.6)
Multiple chemotherapy	157 (55.9)	158 (48.2)	65 (60.8)	72 (59.5)	55 (71.4)	63 (65.0)	37 (38.1)	23 (20.9)
First-line therapy	187 (70.6)	271 (78.8)	70 (68.6)	97 (76.9)	58 (80.6)	89 (87.3)	65 (67.0)	79 (71.8)
Impairment in GA domain								
Functional status								
ADL	83 (29.5)	79 (24.1)	26 (24.3)	29 (24.0)	26 (33.8)	25 (25.8)	31 (32.0)	25 (22.7)
IADL	162 (57.7)	150 (45.7)	57 (53.3)	59 (48.8)	51 (66.2)	50 (51.6)	54 (55.7)	41 (37.3)
Physical performance								
Physical health	220 (78.3)	233 (71.0)	81 (75.7)	81 (66.9)	63 (81.8)	77 (79.4)	76 (78.4)	75 (68.2)
SPPB	241 (85.8)	259 (79.0)	91 (85.1)	91 (75.2)	69 (89.6)	79 (81.4)	81 (83.5)	89 (80.9)
Falls	70 (24.9)	56 (17.1)	21 (19.6)	21 (17.4)	23 (29.9)	17 (17.5)	26 (26.8)	18 (16.4)
TUG	127 (45.2)	120 (36.6)	50 (46.7)	47 (38.8)	36 (46.8)	36 (37.1)	41 (42.3)	37 (33.6)
Comorbidity	197 (70.1)	209 (63.7)	76 (71.0)	76 (62.8)	52 (67.5)	69 (71.1)	69 (71.1)	64 (58.2)
Nutrition								
BMI	33 (11.7)	31 (9.5)	15 (14.0)	14 (11.6)	11 (14.3)	8 (8.3)	7 (7.2)	9 (8.2)
MNA	175 (62.3)	199 (60.7)	79 (73.8)	87 (71.9)	52 (67.5)	65 (67.0)	44 (45.4)	47 (42.7)
Psychological status								
GAD7	41 (14.6)	40 (12.2)	20 (18.7)	15 (12.4)	9 (11.7)	13 (13.4)	12 (12.4)	12 (10.9)
GDS	68 (24.2)	78 (23.8)	22 (20.6)	21 (17.4)	24 (31.2)	29 (29.9)	22 (22.7)	28 (25.5)
Mini-Cog	109 (38.8)	98 (29.9)	47 (43.9)	34 (28.1)	30 (39.0)	29 (29.9)	32 (33.0)	35 (31.8)
BOMC	11 (3.9)	12 (3.7)	5 (4.7)	4 (3.3)	3 (3.9)	4 (4.1)	3 (3.1)	4 (3.6)
Geriatric intervention								
No	120 (42.7)	196 (59.8)	44 (41.1)	62 (51.2)	41 (53.3)	72 (74.2)	35 (36.1)	62 (56.4)
Yes	161 (57.3)	132 (40.2)	63 (58.9)	59 (48.8)	36 (46.8)	25 (25.8)	62 (63.9)	48 (43.6)

Abbreviations: ADL, activity of daily living; BMI, body mass index (calculated as weight in kilograms divided by height in meters squared); BOMC, Blessed Orientation Memory Concentration; GA, geriatric assessment; GAD7, Generalized Anxiety Disorder 7-item scale; GDS, Geriatric Depression Scale; IADL, instrumental activity of daily living; KPS, Karnofsky Performance Scale; MNA, Mini-Nutritional Assessment; NA, not applicable; SPPB, Short Physical Performance Battery; TUG, Timed Up and Go.

^a^ Data are presented as No. (%) of patients.

^b^ Refers to any other solid cancer or lymphoma.

^c^ Includes American Indian or Alaska Native, Asian, or Native Hawaiian or Other Pacific Islander.

**Table 2.** Chemotherapeutic Regimens Commonly Received by Study Participants^a^

Treatment regimen by cancer type	All patients (N = 609)	Standard of care (n = 328)	Primary treatment modification (n = 281)
Lung cancer	174	97	77
Pemetrexed carboplatin with or without pembrolizumab	64 (36.8)	49 (50.5)	15 (19.5)
Paclitaxel carboplatin with or without monoclonal antibody	36 (20.6)	18 (18.5)	18 (23.3)
Etoposide carboplatin	20 (11.4)	9 (9.3)	11 (14.3)
Nab-paclitaxel carboplatin	15 (8.6)	6 (6.2)	9 (11.7)
Gastrointestinal cancer	228	121	107
FOLFOX with or without bevacizumab	64 (28.1)	38 (31.4)	26 (24.2)
Gemcitabine nab-paclitaxel	42 (18.4)	19 (15.7)	23 (21.5)
Capecitabine	23 (10.1)	12 (9.9)	9 (8.4)
FOLFIRI with or without bevacizumab	18 (7.9)	9 (7.4)	9 (8.4)
Genitourinary cancer	55	26	29
Docetaxel with or without prednisone	31 (56.3)	16 (61.5)	15 (51.7)

Abbreviations: FOLFIRI, leucovorin, fluorouracil, and irinotecan; FOLFOX, leucovorin, fluorouracil, and oxaliplatin.

^a^ Data are presented as No. or No. (%) of patients. Data are only reported for commonly received regimens at cycle 1.

### Primary Treatment Modification and Grade 3 to 5 Toxic Effects

**Figure 1.**
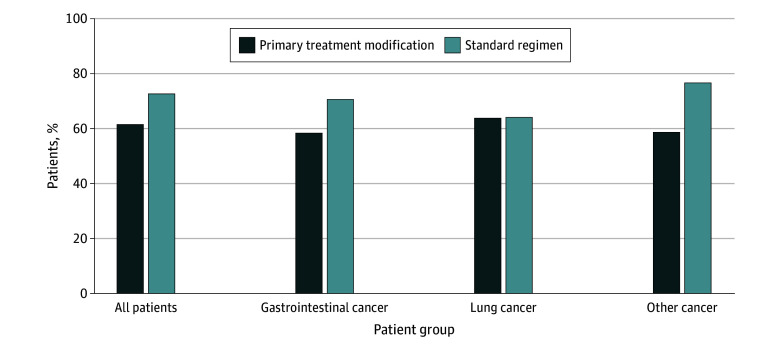
Proportion of Grade 3 or Greater Toxicity Among Patients Who Received Primary Modified Chemotherapy vs Standard-of-Care Regimens The adjusted relative risk (95% CI) was 0.85 (0.77-0.94) for all patients, 0.82 (0.70-0.96) for those with gastrointestinal cancer, 1.03 (0.88-1.20) for those with lung cancer, and 0.82 (0.67-1.00) for those with other cancer types.

Among the study participants, 405 (66.5%) had a grade 3 to 5 toxic effect within 3 months of starting a new chemotherapy regimen (Figure 1). In the unadjusted model, primary treatment modification was associated with a reduced risk of grade 3 to 5 toxic effects (relative risk [RR], 0.86 [95% CI, 0.77-0.96]). After adjustment for covariates, the risk of toxic effects was similar (RR, 0.85 [95% CI, 0.77-0.94]). When stratified by cancer type, primary treatment modification was associated with a decreased risk of toxic effects for patients with gastrointestinal cancer (RR, 0.82 [95% CI, 0.70-0.96]) and other cancer types (RR, 0.82 [95% CI, 0.67-1.00]), but not for patients with lung cancer (RR, 1.03 [95% CI, 0.88-1.20]) (Table 3).

**Table 3.** Risk of Grade 3 to 5 Toxic Effects and Functional Decline Associated With Primary Treatment Modification, Stratified by Cancer Type

Cancer type	Grade ≥3 toxic effects, RR (95% CI)^a^			Functional decline, RR (95% CI)^b^		
	No. of patients	Unadjusted	Adjusted	No. of patients	Unadjusted	Adjusted
Overall	609	0.86 (0.77-0.96)	0.85 (0.77-0.94)	546	0.86 (0.62-1.19)	0.80 (0.67-0.95)
Gastrointestinal	228	0.85 (0.73-0.97)	0.82 (0.70-0.96)	199	1.08 (0.68-1.72)	1.02 (0.66-1.59)
Lung	174	1.06 (0.89-1.26)	1.03 (0.88-1.20)	160	0.81 (0.43-1.55)	0.80 (0.48-1.34)
Other^c^	207	0.75 (0.60-0.95)	0.82 (0.67-1.00)	187	0.74 (0.45-1.22)	0.60 (0.35-1.01)

Abbreviation: RR, relative risk.

^a^ Models adjusted for study group, impaired activities of daily living, and impaired falls.

^b^ Models adjusted for study group, race and ethnicity, previous chemotherapy, multiple chemotherapy, and Karnofsky Performance Scale score.

^c^ Refers to any other solid cancer or lymphoma.

### Primary Treatment Modification and Patient-Reported Functional Decline

**Figure 2.**
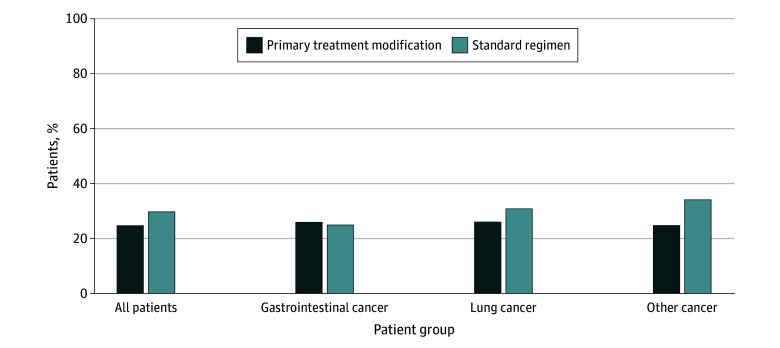
Proportion of Patient-Reported Functional Decline Among Those Who Received Primary Modified Chemotherapy vs Standard-of-Care Regimens The adjusted relative risk (95% CI) was 0.80 (0.67-0.95) for all patients, 1.02 (0.66-1.59) for those with gastrointestinal cancer, 0.80 (0.48-1.34) for those with lung cancer, and 0.60 (0.35-1.01) for those with other cancer types.

Of 546 participants, 153 (28.0%) had patient-reported functional decline (Figure 2). In multivariable analysis, patients who had primary treatment modification had a 20.0% lower risk of functional decline compared with patients who received standard-of-care treatment (RR, 0.80 [95% CI, 0.67-0.95]). When stratified by cancer type, primary treatment modification was associated with a decreased risk of functional decline for patients with lung cancer (RR, 0.80 [95% CI, 0.48-1.34]) and other cancer types (RR, 0.60 [95% CI, 0.35-1.01]), but these decreases were not statistically significant (Table 3).

As an exploratory analysis, we investigated the association between primary treatment modification and changes in IADLs. However, an association between primary treatment modification and IADL decline was not observed (RR, 1.08 [95% CI, 0.92-1.26]).

### Primary Treatment Modification and Composite Adverse Outcome

The composite adverse outcome was favorable in 114 patients (20.8%), intermediate in 333 (61.0%), and poor in 99 (18.1%). Patients who received primary treatment modification, compared with those who received standard-of-care treatment, had 32.0% lower odds (odds ratio, 0.68 [95% CI, 0.48-0.97]) of having a worse composite adverse outcome (ie, worse equaled having an intermediate vs favorable composite adverse outcome or poor vs intermediate; eTable 2 in Supplement 1).

## Discussion

In this secondary analysis of a large cohort of older adults with advanced cancer who were starting a new chemotherapy in the community oncology setting, we observed that patients who received primary treatment modification had improved tolerability (ie, reduced risk of clinician-rated grade 3-5 treatment toxic effects and patient-reported functional decline) compared with these who received standard doses. Furthermore, our findings suggest that this primary treatment modification was associated with tolerability without compromising treatment efficacy. This was determined through a composite adverse outcome, an end point that incorporated clinician-rated toxic effects, patient-reported functional decline, and 6-month survival in 1 outcome measure. To our knowledge, this analysis is one of very few to find an independent association between primary treatment modification and improved treatment tolerability in a cohort of older adults with advanced cancer and aging-related conditions undergoing chemotherapy.

Our findings complement the primary findings of the parent GAP70+ trial by providing an explicit examination of the association between primary treatment modifications and tolerability outcomes among older adults with advanced cancer who are receiving chemotherapy. In contrast with the primary analysis of the GAP70+ trial,^13^ which investigated the impact of GA-guided management on reducing toxic effects in older adults with advanced cancer, our current study specifically explored the association between primary treatment modifications and tolerability outcomes. Although one potential recommendation in the primary GAP70+ study was to employ primary dose reduction in some situations, both the GA and usual care groups included participants who received primary modifications with treatment either through GA recommendations or physician clinical judgment.

In this analysis, we observed that older adults with advanced cancer who received primary treatment modification had a lower risk of grade 3 to 5 toxic effects compared with older adults who received standard-of-care treatment. This finding was consistent among patients with all cancer diagnoses except lung cancer. In a recent randomized clinical trial that included older patients or individuals with frailty with advanced gastroesophageal cancer, those who received reduced doses of chemotherapy (oxaliplatin and capecitabine) had fewer toxic effects.^25^ In the FOCUS II study (n = 459; age range, 35-87 years), the risk of having any grade 3 to 5 toxic effects did not substantially increase with oxaliplatin but was higher with capecitabine than with fluorouracil (40% vs 30%).^26^ Unlike these 2 studies, which included a heterogeneous group of participant ages, our study focused exclusively on older adults with advanced cancer and aging-related conditions, who are typically underrepresented in chemotherapy clinical trials.^1^ It is worth noting that when we conducted stratified analysis by cancer type, we did not find an association between receipt of less intense treatment and reduced risk of chemotherapy-related toxic effects among patients with advanced lung cancer but we observed such an association among patients with gastrointestinal cancer or other cancers. A possible explanation is that patients with lung cancer had a higher risk of mortality compared with patients with other cancer diagnoses.^27^

In this study, 28.0% of older patients (n = 153) with advanced cancer receiving chemotherapy experienced a functional decline (reported by ADL decline) within 3 months of treatment initiation. This percentage is relatively higher compared with other studies that assessed functional decline among older adults with cancer receiving treatment.^17,28^ This higher prevalence of decline may be because our cohort was a vulnerable population, with all patients having advanced cancer and at least 1 geriatric domain impairment. In addition, we demonstrated that patients who received primary modified treatment had a substantially lower risk of ADL decline compared with older adults who received standard regimens. Given that an important treatment goal in the advanced cancer setting is to improve quality of life and patient physical function, this finding suggests that current treatment guidelines may not fit to this population. Similar to grade 3 to 5 toxic effects, the association we observed between primary treatment modification and decreased risk of functional decline was not consistent among different cancer types, suggesting that tolerability outcomes could differ by cancer type and treatment regimens. In an exploratory analysis, we did not find an association between primary treatment modification and IADL decline. One plausible explanation is that IADLs involve complex tasks related to independent living, such as financial management and transportation. The multifaceted nature of IADLs may make them less susceptible to the specific interventions employed in primary treatment modifications, which primarily target toxic effects associated with chemotherapy.^29^

In this study, patients who received primary modified treatment had improved composite adverse outcome compared with patients who received standard doses of treatment. This finding is consistent with prior research suggesting that patients with gastrointestinal cancer have better treatment utility when they receive lower doses or a less intense combination of chemotherapeutic regimens.^25,26^ Composite end points that incorporate tolerability and efficacy measures could be crucial in this population because some patients are willing to tolerate treatment-related adverse events for the potential of survival benefits, whereas others may place a higher value on maintaining functional status and quality of life.^21^

### Strengths and Limitations

A major strength of this study is its inclusion of a population that is historically marginalized in oncology trials—older adults with advanced cancer and aging-related conditions receiving care in community oncology clinics. Additionally, we used robust procedures for ascertainment and review of both exposure data and toxic effects grading by comparing study forms with medical records for all participants.

This study also has several limitations. First, there is a possibility of residual confounding by indication, which may have biased our effect measures of association toward the null (eg, individuals with greater frailty are more likely to receive dose modification; these individuals are also likely to experience toxicity). Second, participants were required to have impairment in at least 1 of the GA domains to enroll in the study, which could lend to extremely healthy older adults being excluded from this analysis. This may limit the generalizability of the study to a small extent. Third, the study included multiple heterogeneous cancer types that may have different options for primary treatment modifications, which may lead to variation in toxic effects by cancer type. Fourth, an additional consideration pertains to the subgroup analyses conducted by cancer type. Although these analyses were exploratory and aimed to elucidate potential variations in the associations between primary treatment modification and tolerability outcomes across different cancer types, we acknowledge that the study was not explicitly powered for this purpose. Thus, the findings from these subgroup analyses should be interpreted with caution and are presented for the purpose of guiding future research efforts. Fifth, our study participants were primarily White and English speakers. Thus, our findings may not be generalizable to other groups.

## Conclusions

In this cohort study of older adults with advanced cancer who were initiating new chemotherapy in the community oncology setting, we observed that patients undergoing primary treatment modification experienced improved tolerability, indicated by a reduced risk of clinician-rated grade 3 to 5 treatment toxic effects and patient-reported functional decline, compared with those receiving standard doses. In addition, our findings illustrate that primary treatment modification improved patient tolerability without compromising treatment efficacy, as assessed through a composite adverse outcome measure. This information can help oncologists to choose the optimal drug regimen, select a safe and effective initial dose, and undertake appropriate monitoring strategies to manage the clinical care of older people with advanced cancer. Future trials are needed to confirm these findings, focusing on different cancer types, treatment regimens, and specific types of treatment modifications to better understand and optimize cancer treatment decision-making in older adults with advanced cancer and aging-related conditions.

## Notes

Supplement 1. **eTable 1.** Description of Geriatric Assessment Domains and Tests **eTable 2.** Association Between Primary Treatment Modification and Composite Adverse Outcome Measure **eFigure 1.** Study Schema **eFigure 2.** Breakdown of Primary Treatment Modification Forms (n = 281)

Supplement 2.
Data Sharing Statement
