# Supplementary material for: Primary Treatment Modification and Treatment Tolerability Among Older Chemotherapy Recipients With Advanced Cancer
Source: JAMA Netw Open. 2024 Feb 15;7(2):e2356106. doi: 10.1001/jamanetworkopen.2023.56106 (PMC10870189; doi:10.1001/jamanetworkopen.2023.56106)
Supplement: Supplement 1. — eTable 1. Description of Geriatric Assessment Domains and Tests eTable 2. Association Between Primary Treatment Modification and Composite Adverse Outcome Measure eFigure 1. Study Schema eFigure 2. Breakdown of Primary Treatment Modification Forms (n = 281) [file jamanetwopen-e2356106-s001.pdf]

Supplementary Online Content

Mohamed MR, Rich DQ, Seplaki C, et al. Primary treatment modification and treatment tolerability among older chemotherapy recipients with advanced cancer. *JAMA Netw Open*. 2024;7(2):e2356106. doi:10.1001/jamanetworkopen.2023.56106

eTable 1. Description of Geriatric Assessment Domains and Tests

eTable 2. Association Between Primary Treatment Modification and Composite Adverse Outcome Measure

eFigure 1. Study Schema

eFigure 2. Breakdown of Primary Treatment Modification Forms (n = 281)

This supplementary material has been provided by the authors to give readers additional information about their work

© 2024 Mohamed MR et al. *JAMA Network Open.*

eTable 1. Description of Geriatric Assessment Domains and Tests

Domains	Elements of assessment	Descriptions (impairment)
Physical performance	Timed "Up and Go"	Assess mobility over 3 meters; longer time indicates worse performance (>13.5 seconds)
	Short Physical Performance Battery	Assess balance, gait speed, and strength; higher score indicates better performance (≤ 9 points)
Functional status	ADL	Assess difficulty with the following 6 activities: bathing, dressing, eating, getting in and out of bed/chairs, walking, toileting (options: yes/no) (Any deficit (yes))
	Instrumental ADLs	Assess independence in the following 7 activities: using the telephone, transportation, shopping, preparing meals, doing housework, taking medicine, managing money (options: without help, with some help, completely unable to) (Any deficit (yes))
Comorbidity	OARS Comorbidity	Assess the presence of 13 illnesses (e.g. other cancer or leukemia, arthritis, glaucoma) as well as hearing and visual impairments, and how much each problem interferes with his/her activities (options: not at all, somewhat, a great deal) (Patient answered "yes" to 3 illnesses OR answered that 1 illness interferes "a great deal" (including eyesight and hearing))
Nutrition	Body Mass Index	Divide weight in kilograms by height in meters squared (< 21 kg/m)
	Mini Nutrition Assessment	Assess nutritional status using 6 items; lower score is worse (range 0-14 points) (≤ 11 points)
Psychological status	Geriatric Depression Scale	Assess depression using 15 items; higher score is worse (≥ 5 points)
	Generalized Anxiety Disorder-7 item scale	Assess anxiety using 7 items; higher score is worse, range 0-21 points (≥ 10 points)

Abbreviations: ADL, activity of daily living; OARS, Older Americans Resources and Services

© 2024 Mohamed MR et al. *JAMA Network Open.*

eTable 2. Association Between Primary Treatment Modification and Composite Adverse Outcome Measure

	**Adjusted odds ratio**	**95% confidence interval**	**p-value**
**Overall (N=546)**	0.68	0.48-0.97	0.03
**Gastrointestinal (N=199)**	0.62	0.34-1.04	0.10
**Lung Cancer (N=160)**	1.06	0.56-2.02	0.86
**All cancer types except lung (N=386)**	0.55	0.36-0.85	<0.01

© 2024 Mohamed MR et al. *JAMA Network Open.*

eFigure 1. Study Schema

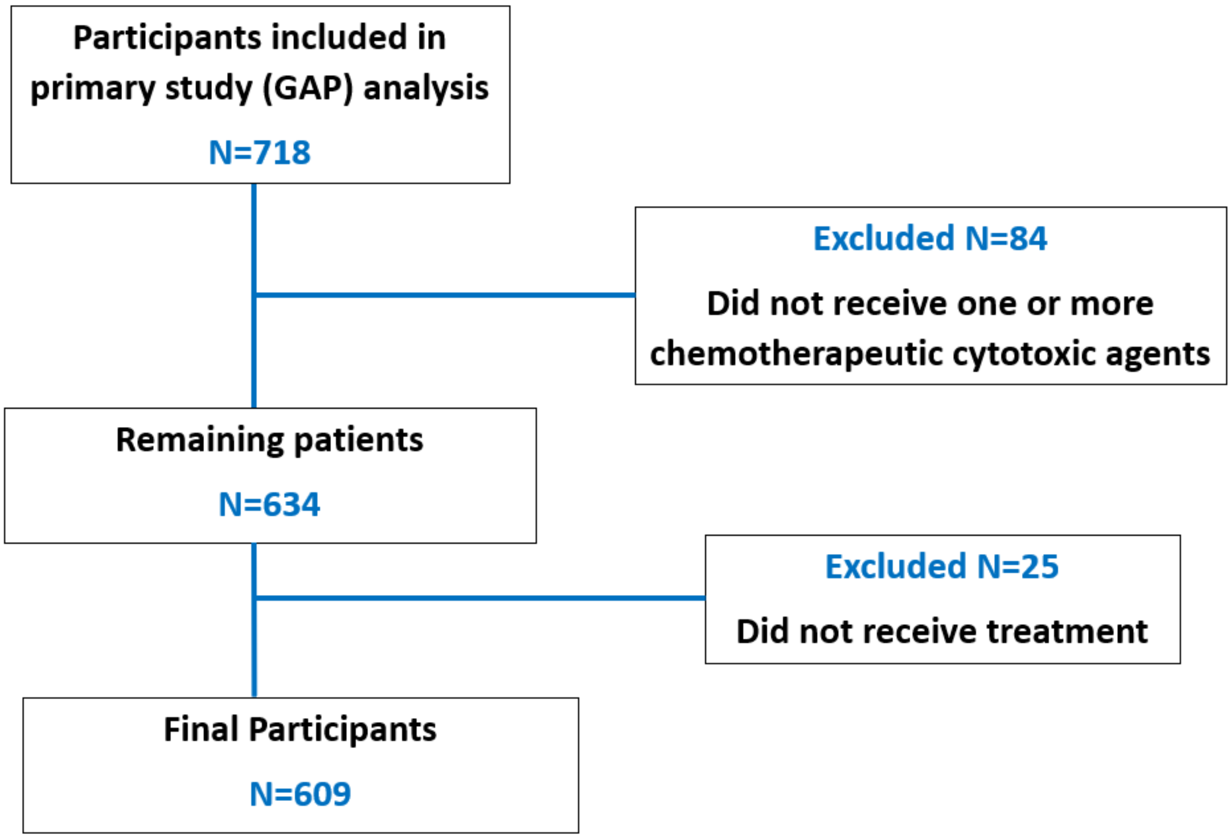

© 2024 Mohamed MR et al. *JAMA Network Open.*

eFigure 2. Breakdown of Primary Treatment Modification Forms (n = 281)

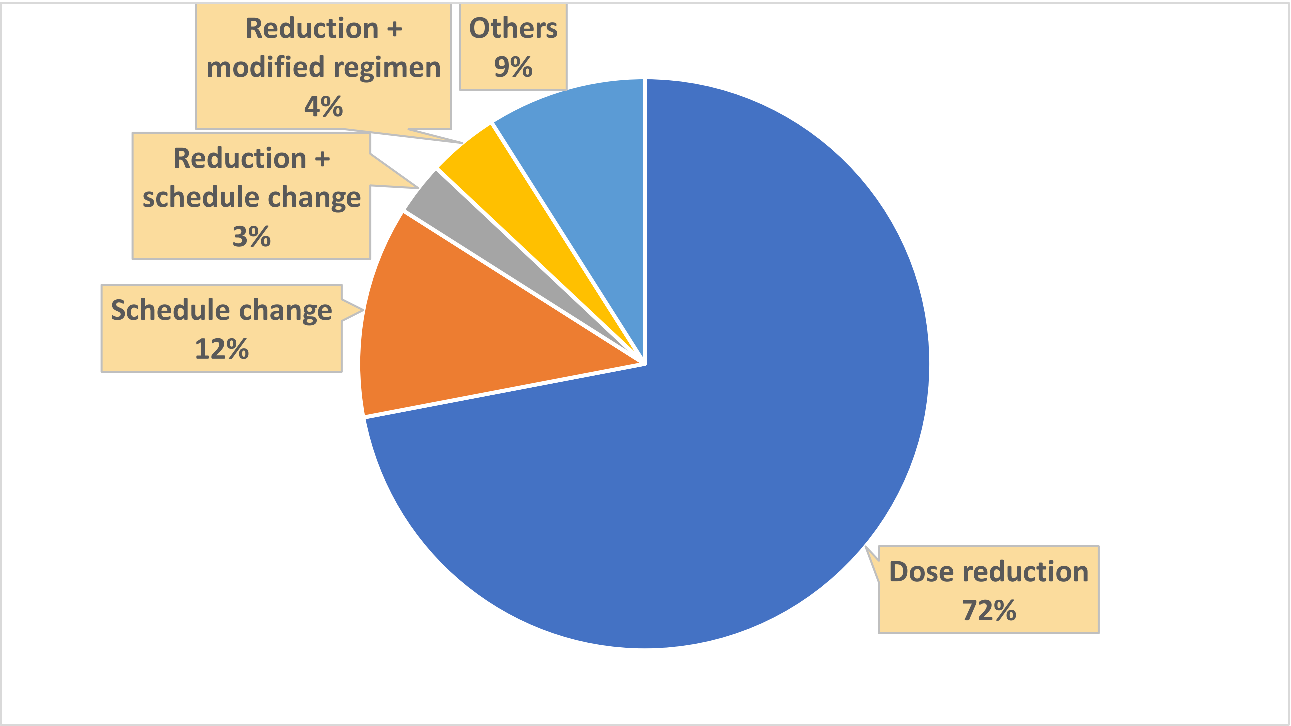

© 2024 Mohamed MR et al. *JAMA Network Open.*
