# Supplementary material for: Primary Treatment Modification and Treatment Tolerability Among Older Chemotherapy Recipients With Advanced Cancer
Source: JAMA Netw Open. 2024 Feb 15;7(2):e2356106. doi: 10.1001/jamanetworkopen.2023.56106 (PMC10870189; doi:10.1001/jamanetworkopen.2023.56106)
Supplement: Supplement 2. — Data Sharing Statement [file jamanetwopen-e2356106-s002.pdf]

Data Sharing Statement

Mohamed. Primary Treatment Modification and Treatment Tolerability Among Older Chemotherapy Recipients With Advanced Cancer. *JAMA Netw Open*. Published February 15, 2024. doi:10.1001/jamanetworkopen.2023.56106

Data
Data available: Yes
Data types: Data (not involving human participants)
How to access data: mostafa_mohamed@urmc.rochester.edu
When available: With publication

Supporting Documents
Document types: None

Additional Information
Who can access the data: researchers whose proposed use of the data has been approved
Types of analyses: any purpose
Mechanisms of data availability: with a signed data access agreemen
